# Alkyne-Azide “Click” Chemistry in Designing Nanocarriers for Applications in Biology

**DOI:** 10.3390/molecules18089531

**Published:** 2013-08-08

**Authors:** Pramod K. Avti, Dusica Maysinger, Ashok Kakkar

**Affiliations:** 1 Montreal Heart Institute, Research Center, 5000 Bélanger Est, Montréal, QC H1T 1C8, Canada; 2 Institute of Biomedical Engineering, École Polytechnique de Montréal, Montreal, QC H3C 3A7, Canada; 3 Department of Chemistry, McGill University, 801 Sherbrooke St. W. Montréal, QC H3A 0B8 Canada; 4 Department of Pharmacology and Therapeutics, McGill University, 3655 Promenade Sir-William-Osler, Montreal, QC H3G 1Y6, Canada

**Keywords:** click chemistry, copper catalyzed alkyne-azide cycloaddition, drug delivery, lipid bodies, mitochondria

## Abstract

The alkyne-azide cycloaddition, popularly known as the “click” reaction, has been extensively exploited in molecule/macromolecule build-up, and has offered tremendous potential in the design of nanomaterials for applications in a diverse range of disciplines, including biology. Some advantageous characteristics of this coupling include high efficiency, and adaptability to the environment in which the desired covalent linking of the alkyne and azide terminated moieties needs to be carried out. The efficient delivery of active pharmaceutical agents to specific organelles, employing nanocarriers developed through the use of “click” chemistry, constitutes a continuing topical area of research. In this review, we highlight important contributions click chemistry has made in the design of macromolecule-based nanomaterials for therapeutic intervention in mitochondria and lipid droplets.

## 1. Introduction

In 2001 Sharpless introduced the concept of “click chemistry”, one of the most versatile and modular approaches to couple two reactive partners in a facile, quick, selective, reliable and high yield reaction under mild conditions [1]. Since then click chemistry has become one of the most common and reliable methods to link molecules covalently, and it finds applications in a variety of disciplines including the chemistry of nanomaterials, chemical biology, drug delivery, and medicinal chemistry [2,3,4,5,6,7]. The inherent properties of click chemistry are also characteristic of “green chemistry” reactions. Although the 1,3-dipolar Hüisgen cycloaddition of azides with terminal alkynes was discovered in 1963, the copper catalyzed alkyne-azide cycloaddition (CuAAC) has become increasingly popular in the last decade. One of the reasons for this is that the traditional 1,3-dipolar Hüisgen cycloaddition takes place at high temperatures [8,9]. In addition, CuAAC reaction can be performed in a variety of solvents such as water, ethanol or *tert*-butyl alcohol, *etc*. [10]. Other advantages of CuAAC reaction include its efficiency under physiological conditions, and its chemo-selectivity, which allows labeling of functional biomolecules such as peptides, proteins, nucleic acids, polysaccharides, *etc*. [11]. It has been suggested that copper catalyst used in the reaction could have some adverse effects related to its toxicity [12]. Alternatives to the use of Cu catalysts in the click reaction, such as metal free cycloaddition reactions [13], and the use of other metals in promoting this reaction [14], have sparked increasing interest in the scientific community. This review aims to summarize the elegant use of alkyne-azide click chemistry in conjugation and designing products, especially intended for applications in biology. We specifically highlight the design of nanocarriers for the delivery of therapeutic agents to mitochondria and lipid droplets, cell organelles of considerable importance in preventing a variety of pathological disorders.

## 2. Copper Catalyzed Alkyne-Azide Cycloaddition (CuAAC)

**Scheme 1:**
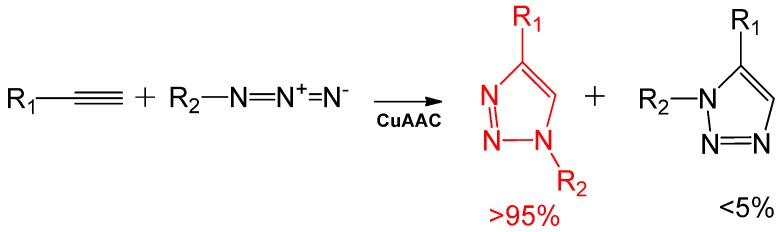
Copper-catalyzed alkyne-azide cycloaddition.

The 1,3-dipolar cycloaddition of azides with alkynes was first discovered by Hüisgen in 1963. However, it did not attract much interest until it was demonstrated that this high temperature reaction could also be carried out under mild conditions using Cu(I) as the catalyst, and with tremendous regio-selectivity (Scheme 1). This was discovered simultaneously and independently by Meldal and his group in Denmark, and Fokin and Sharpless in USA [9,10,15,16]. The coordination of Cu(I) to alkynes in an aqueous solution forming a copper-acetylide intermediate is an exothermic reaction. The azide binds to this Cu (I)-acetylide intermediate forming a six membered Cu(III)-metallacycle [10]. Subsequently, the triazole ring formation is very rapid [17], and the cycloaddition product is chemically inert or stable towards redox reactions, has strong dipole moment, hydrogen bond accepting ability and aromatic character [18]. Experimental and computational studies have shown that Cu(I) coordinates to the alkynes through polynuclear Cu(I) intermediates [17,19,20,21,22,23]. Recently, a detailed mechanism has been elucidated by Fokin and his colleagues [24]. The advantages of this alkyne-azide coupling reaction include an almost quantitative conversion, the robust nature of the products, biomolecular ligation, *in vivo* tagging [25,26,27,28], and use in the synthesis of linear polymers [29,30].

The CuAAC reaction has been successfully introduced in many different scientific areas, and its potential has been demonstrated in materials chemistry [31], dendrimer build-up [32], polymers [33,34], nanoparticle synthesis [35] and interlocked molecules [36,37]. In dendrimer chemistry, CuAAC was used not only for the convergent [38] and divergent build-up [39,40], but also for the dendrimer functionalization, and introduction of multiple functionalities into the macromolecular architecture [32,41,42,43,44,45,46]. For biomedical applications the use of Cu in the reaction and its retention post-synthesis, poses potential toxicity risks, and thus could limit the use of this method for products intended for biology [12]. Copper metal is added in catalytic amounts in the reaction, and is subsequently removed after reaction by adding chelating ligands such ethylenediaminetetraacetic acid (EDTA). However, considering the potential adverse effects, even at picomolar levels, Cu-free click strategies have been developed recently which reduce the risk of transition metal related toxicity issues [47,48,49,50]. Copper-free reactions described by Bertozzi and her colleagues as strain promoted alkyne-azide cycloaddition (SPAAC) date back to the work done by George Wittig, who described the exothermic cycloaddition of cyclooctyne with phenyl azide leading to triazoles [51]. These reactions showed immense potential *in vivo* [52,53], and have been extended to label peptides [54], DNA [55,56] and lipids [57], to cross-linked hydrogels [58], polymers [59,60] and photodegradable star polymers [61]. The other type of SPAAC reactions include cycloadditions between strong 1,3-dipoles with enhanced reactivity such as nitrile oxides, nitrile imines and nitrones with unsaturated hydrocarbons [62,63,64], applicable in DNA bioconjugation reactions [65,66,67,68]. The following sections provide several examples of click chemistry reactions employed in developing nanocarriers for targeted drug delivery to cellular organelles.

## 3. Drug Delivery

Tremendous effort has been devoted to the development of nanocarriers for the efficient delivery of therapeutic agents to the targeted site [69]. In this regard macromolecules have offered tremendous potential [70], but such nanodelivery systems have to meet stringent requirements if they are to be employed for drug delivery [71,72]. The macromolecule based nanocarriers used for this purpose should be non-cytotoxic, remain intact prior to reaching the target site, and enhance the effectiveness of the selected drug. Although, significant efforts have been made in assembling macromolecule based nanocarriers using a variety of synthetic methodologies, challenges still remain in introducing multiple functions into a single platform. Click chemistry has offered new ways of developing nanomaterials [60,73,74], particularly those with multiple functional groups and architecture [75]. These moieties can be introduced within the nanocarrier architecture with high precision. Such nanoarchitectures have been exploited as suitable carriers for therapeutic agents and fluorescent labels to deliver them to specific cells, cellular organelle, to either prevent cell death [76] or visualize them with or without drug delivery. A number of strategies to target cells with drugs had been adopted earlier, and these include carbodiimide, thiol-maleimide and biotin-avidin coupling to biomolecules [77]. As already mentioned, recent progress in click chemistry has allowed coupling reactions to be carried out under mild conditions, and in an aqueous medium with negligible unwanted toxic bye-products [1]. Using copper free alkyne-azide coupling, one can link a variety of peptides, antibodies and drugs to biocompatible synthetic macromolecules that have been specifically targeted to the cells [78,79,80]. Considering the focus of this review article, the following sections provide a few examples of nanodelivery systems targeting cell organelles, specifically mitochondria and lipid bodies (LBs). 

### 3.1. Mitochondria

Mitochondria, cellular power plants, play pivotal homeostatic role in cellular functions such as cellular signaling, growth and differentiation, cell cycle regulation, electron transport, calcium storage and cellular death [81,82]. Mitochondrial dysfunction is implicated in a variety of pathological disorders such as aging, ischemia-reperfusion, cardiac disorders, neurodegenerative and neuromuscular diseases, obesity, and genetic disorders [83,84,85,86]. One of the major causes of damage in these conditions is the generation of mitochondrial reactive oxygen species [87]. N-acetylcysteine, α-lipoic acid and coenzyme Q10 (CoQ10) are some of the antioxidant therapeutics that have shown promise inneurodegenerative diseases [88,89,90]. CoQ10 or ubiquinone is a naturally occurring lipid-soluble vitamin-like benzoquinone derivative with 10 monounsaturated *trans*-isoprenoid units in the side chain, and acts as a cofactor for mitochondrial complexes I–III for the generation of ATP [91,92]. It is found in the inner mitochondrial and cellular membranes, blood and in high and low-density lipoproteins [93]. Some of the main disadvantages of selective drugs are their hydrophobicity, stability, bioavailability, inability to cross the membrane barriers and selective accumulation in the multi-membrane barrier organelles located in the cytoplasm, such as mitochondria. Targeting mitochondria with a variety of bioactive molecules and drugs is one strategy to overcome some of these hurdles [83]. Many strategies had been reported earlier for the delivery to mitochondria such as use of lipophilic cations [94,95,96], protein-nucleic acid [97], peptide-nucleic acid [98,99,100,101], protein and RNA [102,103,104], and peptides [105,106]. The efficient and organelle specific delivery of therapeutics continues to be a topical area, and nanocarriers based approaches are emerging. Unlike cellular targeting, the prerequisite for mitochondrial targeting includes the use of drug modifications or encapsulation into nanocarriers such as dendrimers. This would help not only cross several membrane barriers, but also have high accumulation in these organelles. The other advantage of using the nanocarrier systems is their ability for site specific targeting with improved efficacy and reduced toxicity [89,107,108,109].

**Figure 1:**
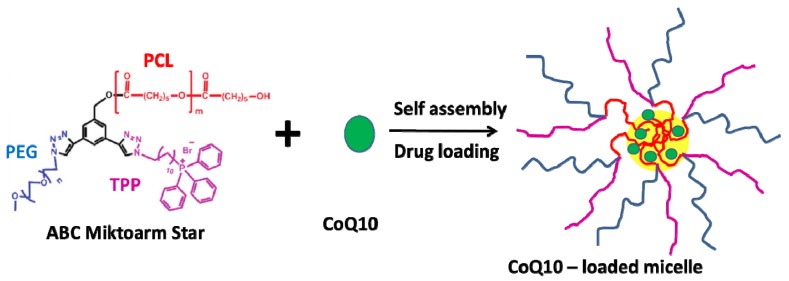
Self-assembly of the ABC miktoarm star polymer, and loading of CoQ10 into the resulting micelles.

Recently, our group synthesized multifunctional nanocarriers based on miktoarm polymers of the type ABC [A = poly(ethylene glycol (PEG), B = polycaprolactone (PCL), and C = triphenyl-phosphonium bromide (TPPBr)], for targeting mitochondria and to deliver coenzyme Q10 (CoQ10) [110] (Figure 1). The delivery system was synthesized using a combination of click chemistry with ring-opening polymerization, and subsequently self-assembled into nanosized micelles loaded with CoQ10. The loaded micelles of size 25–60 nm with a capacity of more than 70 wt% for CoQ10 were stable in solution for 3 months. The high loading efficiency in these clicked polymers, unlike other carrier systems for CoQ10 [111,112], resulted in low drug loss, and showed high efficacy as nanotherapeutics against oxidative stress-induced cell damage [110]. 

**Figure 2:**
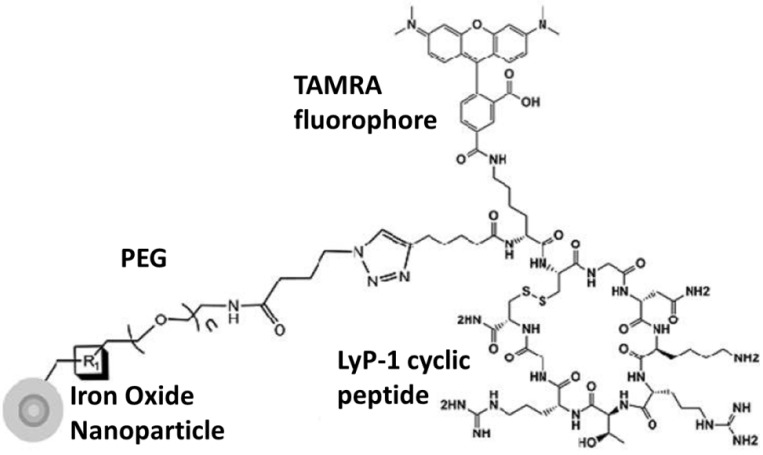
Superparamagnetic iron oxide nanoparticle labeled with fluorochrome (TAMRA) and LyP-1 cyclic peptide (Reprinted with permission, [87]).

Targeting mitochondria with peptides is another approach in which CuAAC was used to conjugate a cyclic tumor targeting peptide LyP-1 (CGNKRTRGC) to iron oxide nanoparticles with azido-functionalized PEGylated groups [113] (Figure 2). This peptide binds to a mitochondrial peptide p32 that is overexpressed in tumor cells, macrophages and endothelial cells. This clicked product showed blood stability for >5 h *in vivo* which allowed its accumulation in the tumor interstitium. These nanocarriers could be specifically targeted to the tumor sites, providing a platform for the treatment by magnetic hyperthermia. Such a treatment is based on generation of heat by magnetic nanoparticles exposed to the alternating magnetic fields [114]. 

**Figure 3:**
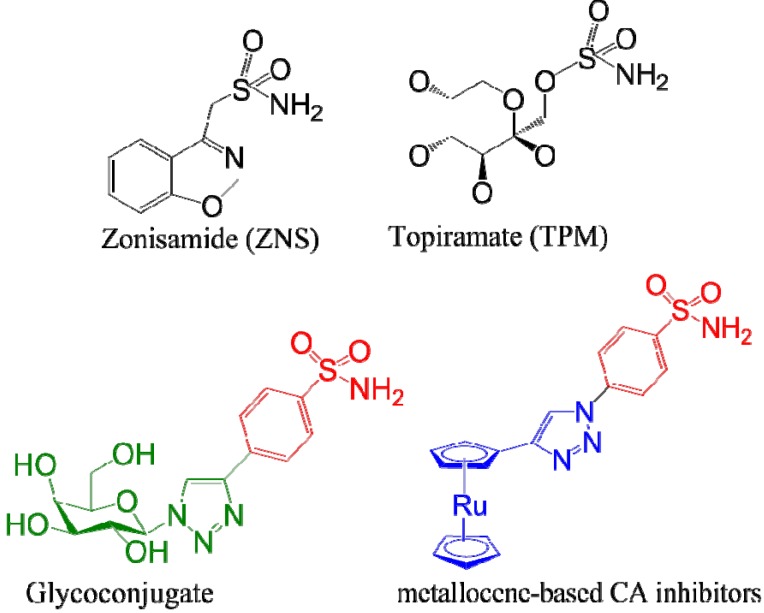
Glycoconjugate and metallocene-based CA inhibitors. The groups in red indicate the CA recognition (Zinc binding motif), green show the sugar-triazole tail and blue the metallocene-triazole tail.

Another interesting approach to target mitochondrial enzyme carbonic anhydrases (CA-VA and VB) has been recently proposed. This approach was suggested as a new platform for the development of anti-obesity treatment strategies [115,116]. Carbonic anhydrases are ubiquitously expressed metallo(zinc)enzymes, involved in the gluconeogenesis, lipogenesis, ureagenesis and tumorigenicity [117]. The mitochondrial CA isozymes are involved in maintaining the availability of HCO_3_^−^ for the formation of pyruvate from citrate. The pyruvate thus formed is translocated to the cytoplasm and is involved in the *de novo* lipogenesis [117]. Different strategies have been proposed in the synthesis of CA inhibitors (CAI). Weight loss was observed during the treatment with zonisamide (ZNS) and topiramate (TPM), containing sulfonamide (–SO_2_NH_2_) and sulfamate–(-OSO_2_NH_2_) moieties (Figure 3). These moieties enable an interaction with the zinc binding sites thereby inhibiting the CA function [118].

ZNS and TPM structures prompted Supuran and colleagues to investigate and synthesize new CA inhibitors. Using ‘click chemistry’ approach, both the glycoconjugates and metallocene-based CA inhibitors were prepared where benzenesulfonamide moiety was linked to sugar or metallocene tail through 1,2,3-triazole group [119,120]. These compounds were effective as CA inhibitors. Additional 10 small molecules of CAI were synthesized using CuAAC method of the azido-benzenesulfonamide fragment with different substituents of phenyl acetylenes [115]. CA catalyzed CO_2_ hydration assay was performed to compare the inhibition potency of ZNS, TPM, and all the 10 aryl triazole inhibitors. These triazole inhibitors were stronger inhibitors of mitochondrial CA isozymes VA and VB as compared to ZNS and TPM. 

Other strategies including incorporation of mitochondrion-targeting peptides have been used to deliver drugs to this organelle [121,122]. More recently an interesting new approach was taken by Dhar’s group [123]. This study showed the versatility of biodegradable high density lipoprotein-nanoparticles for detection of plaques by targeting the collapse of the mitochondrial membrane potential. The same study described a rationally designed mitochondria-targeted polymeric nanoparticle (NP) system and its optimization for efficient delivery of various mitochondria-acting therapeutics by blending a targeted poly(d,l-lactic-co-glycolic acid)-block (PLGA-b)-poly(ethylene glycol) (PEG)-triphenylphosphonium (TPP) polymer (PLGA-b-PEG-TPP) with either nontargeted PLGA-b-PEG-OH or PLGA-COOH. An optimized formulation was identified through *in vitro* screening of a library of charge- and size-varied NPs. A programmable NP platform for the diagnosis and targeted delivery of therapeutics for mitochondrial dysfunction-related diseases was also described [123]. The same group also showed how *in situ* light activation amplifies the host immune responses when NPs deliver the photosensitizer to the mitochondria, and opening up the possibility of using mitochondria-targeted-NP treated, light activated, cancer cell supernatants as possible vaccines [124]. An overview of strategies to target organelles by exploiting different nanotechnological tools was recently reported [125].

### 3.2. Lipid bodies (LB)

Lipid bodies (LBs) are cytoplasmic organelles which have been historically considered cellular storage sites. LBs are phylogenetically conserved and ubiquitous organelles with many cellular functions [126,127,128,129]. More recently, they have been recognized as dynamic, communicating with different organelles including mitochondria [130,131]. Different stressful conditions resulting in mitochondrial damage can lead to LB accumulation. The endoplasmic reticulum (ER) is a major intracellular compartment involved in neutral lipid synthesis and LB biogenesis. Our studies indicated that mitochondrial disruption in cells exposed to cytotoxic nanocrystals is accompanied by LB accumulation [132]. LB accumulation commonly results from inhibition of mitochondrial fatty acid β-oxidation [133]. 

Accumulation of LBs in leukocytes and macrophages follows their stimulation with pro-inflammatory agents including bacterial endotoxins (e.g., lipopolysaccharide from Gram negative bacteria) is well recognized [132,134]. Due to their prominence in inflammatory leukocytes, LBs are considered to be structural markers of inflammation. Therefore, pharmacological modulation of LB biosynthesis and composition presents an attractive strategy to correct LB abnormalities in different pathologies. 

**Figure 4:**
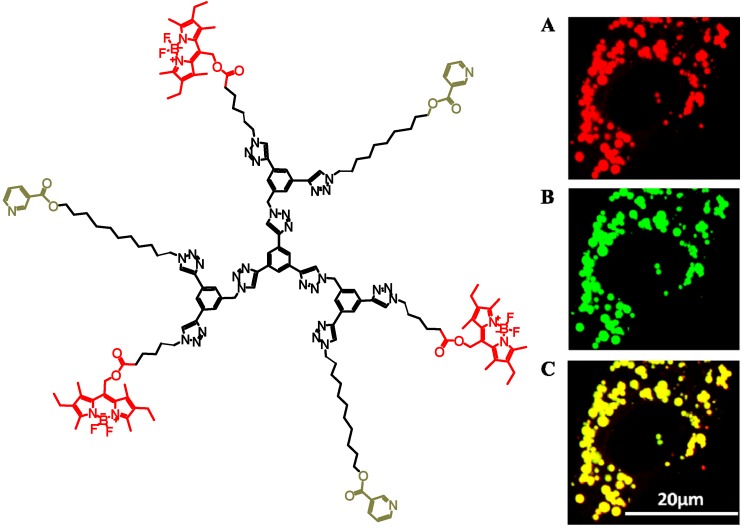
Dendrimer with covalently linked BODIPY dye, and confocal images A: Red BODIPY conjugated dendrimer; B: Lipid droplets labeled with Green BODIPY; and C: Overlay of A and B.

To specifically target LBs, Kakkar and Maysinger developed a macromolecule-based delivery system using click chemistry [135]. The goal was to deliver niacin (and eventually other lipid-modifying drugs) to LBs by means of dendrimer and miktoarm polymer-based nanocarriers, in order to inhibit the activity of LB-localized enzymes. The construct associated with LBs, but the activities of different lipid synthesizing enzymes were not determined. The data from analyses of enzymatic activities contributing to lipid processing in association with LBs would provide valuable information for the development of disease-modifying therapeutics. The delivery vehicles were constructed on building blocks with orthogonal functionalities which allow the introduction of multi-tasking units one at a time [136]. The dendrimer based nanocarrier (Figure 4) was synthesized by clicking a building block with an arm with protected acetylene and another with a long chain alcohol, on to 1,3,5-triethynylbenzene [105]. Niacin, (vitamin B3) was then covalently attached by linking through the long chain alcohol leading to the formation of an ester bond. Upon cleaving the latter bond by cellular esterases, niacin is released from its nanocarrier. The acetylene unit on the delivery vehicle was then deprotected, and BODIPY-azide was covalently linked using CuACC. The detection of the polymers at the subcellular level was made possible by the linked lipophilic fluorescent, non-polar dye, Bodipy 493/503. In order to assess the efficacy of the niacin-conjugated carriers as a LB targeting drug delivery system, the colocalization of nanocarrier with LBs was assessed by confocal microscopy. The intracellular LBs were labeled with the fluorescent dye Bodipy 493/503 (green) which selectively labels neutral lipids, and the nanocarriers were labeled with red fluorescent dye. Within seconds, the carriers entered the cell’s cytosol and localized on cytoplasmic LBs, revealing yellow regions corresponding to the LBs (Figure 4). The achievement of specific targeting of niacin-macromolecules to LBs described by Maysinger and Kakkar [135] may open new means of drug delivery in pathologies characterized by abnormalities in lipid metabolism and lipid storage, such as metabolic steatosis, obesity and atherosclerosis.

We have also synthesized delivery vehicles using click reaction for linkage of α-lipoic acid (LA) and Bodipy [136] to target cellular lipid droplets. Lipoic acid, an essential cellular cofactor, antioxidant, chelating agent and transcription factor regulator [137], is easily taken up by cells and reduced to dihydrolipoic acid which is more effective than LA. LA was covalently linked to the dendrimer which improved its intracellular retention, and showed therapeutic effectiveness. We have recently designed and prepared dendrimers using a combination of CuAAC with Diels-Alder (DA) click reaction in which LA was linked to the periphery of the dendrimer [138]. [4+2] cycloaddition of a diene with a dienophile, popularly known as the Diels-Alder reaction is another highly advantageous reaction belonging to the “click chemistry” family [139]. This alternative strategy has provided an additional straightforward route to the construction of a variety of macromolecules or their functionalization at the periphery. One important aspect of this cycloaddition is its thermal reversibility, commonly referred to as the retro Diels-Alder reaction [140]. We have taken advantage of this property and designed a thermosensitive dendrimer based nanodelivery system for Lipoic acid. The dendrimer was synthesized using two different bifunctional units having an azide with two furan rings (AzFu_2_) and flexible acetylene arms. LA was then clicked via DA reaction to the peripheral furan moieties on the dendrimer. The dendrimer was non cytotoxic and the drug was released from it via a retro-Diels-Alder reaction at 37–42 °C [138]. This study demonstrated that retro-Diels-Alder reaction on moieties covalently linked to macromolecules based nanocarriers, can be carried out at ambient temperatures. It offers a highly exciting platform in which dendrimer-drug conjugates can be assembled using this “click” strategy, and anti-inflammatory agents covalently bound by Diels-Alder click reaction can be released, in a controlled manner, under physiological and pathological range of temperatures.

Another example of Diels-Alder “click” chemistry used for the delivery of drugs through peptides was reported by Braun’s group [141]. It involved the delivery of a cytotoxic drug temozolomide (TMZ) using cyclic-RGD-ligand as cargo to target α_v_β_3_ integrin receptor for cancer. The cytotoxic drug TMZ was ligated to the cRGD-ligand using Diels Alder reaction with inversion electron demand [141]. For evaluating the cellular location of this click product, a fluorescent tag dansyl was ligated. The cRGD-TMZ-dansyl complex when treated to MCF-7 cancer cells effectively binds on to the cell membrane which expresses high levels of α_v_β_3_ integrin. This study also reports that the above click product selectively kills cancer cells with high efficacy as compared to only the TMZ drug treatment. In this section we provide examples of click chemistry to generate nanostructures targeting selected cellular organelles. Methodological details on imaging organelles have been recently reviewed [142].

### 3.3. Click Chemistry for the Synthesis of Nanocarriers with Anti-Inflammatory Properties

**Figure 5:**
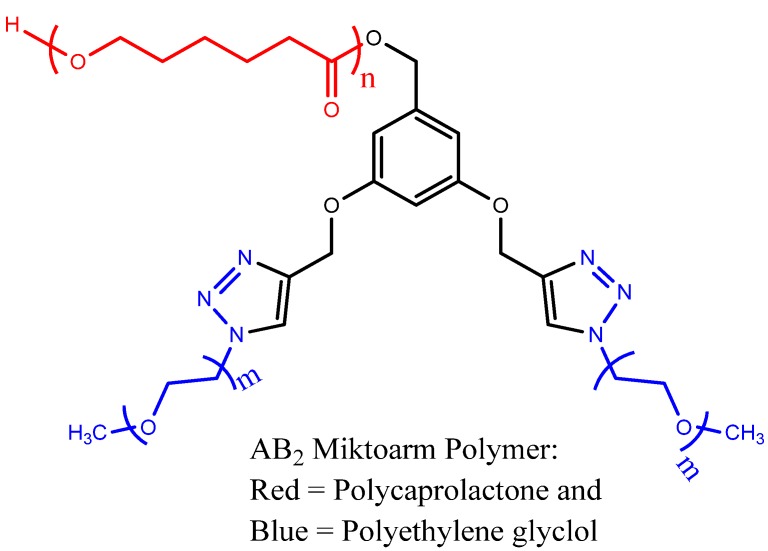
Structure of AB_2_ miktoarm star polymer.

Nimodipine (NIM), an active calcium channel blocker, is a hydrophobic drug with poor aqueous solubility. It is used in the prevention and treatment of cerebral vasospasm and ischemia, both of which occur during the subarachnoid hemorrhage or cerebral bleeding [143]. Clinically, NIM has limited use because its oral administration leads to rapid clearance through liver, making its availability as low as 10%. Because of low water solubility and a need for solubilizing mediator, its administration can cause local adverse effects [109]. Recently, we reported the synthesis of AB_2_ type miktoarm polymer (A = polycaprolactone (PCL); B = polyethylene glycol (PEG)) based nanocarrier using click chemistry (Figure 5), for improving water solubility and delivery of nimodipine (NIM) [109]. The polymer was constructed on a core containing two alkynyl moieties facilitating the click reaction for linking azide terminated PEG, and one alcohol group for ring-opening polymerization of caprolactone. The polymer (PEG775_2_ - PCL5800) assembles into micellar structures into which NIM was loaded with high efficiency (up to 78%), using a co-solvent evaporation method. It led to ~200 fold increase in the aqueous solubility of NIM. The micelles loaded with NIM reduced LPS-induced nitric oxide and pro-inflammatory cytokines (IL-1β and TNF-α) in microglial cells, through a slow release delivery mechanism from the polymers. Intriguingly, the polymers themselves (without drug loading) have shown protection of microglial cells from the LPS stress, indicating anti-inflammatory role of these polymers towards neuro-inflammation.

Recently, Choi and Maysinger showed monitoring of the effectiveness of nanotherapeutics *in vivo* in an animal model of ischemic brain injury [144]. Progression of neurodegeneration and regression of the ischemic lesion upon therapeutic interventions were determined in real-time using luminescent nanocrystals. Intranasal administration of anti-inflammatory nanotherapeutics (micelle-incorporated nimodipine or minocycline) was effective in preventing lesion progression as evidenced by the smaller lesion volumes and by significantly improved motor functions.

It was proposed earlier that the 4th generation poly(amidoamine) (PAMAM) based dendrimers could themselves exert anti-inflammatory effects in the absence of any anti-inflammatory drugs [145]. These studies motivated us to investigate the anti-inflammatory role of our low generation dendrimers (DG0 and DG1) with surface terminal acetylene and hydroxyl groups. These the dendrimers synthesized using “click” chemistry showed inhibition of LPS-induced nitric oxide (NO) and prostaglandin E2 (PGE_2)_ release in the microglial cells without affecting the cell viability and mitochondrial metabolic activity [6]. NO and PGE_2_ are synthesized by the action of iNOS and COX-2. We subsequently investigated if the dendrimers were directly interacting with these enzymes. Computer assisted molecular docking studies were performed to understand their interaction with the enzymes. The results suggest that the low generation dendrimers with terminal -OH functionalities directly interact with the iNOS and COX-2 enzymes active sites more favorably than their acetylene terminated functional groups. The anti-inflammatory effect is mainly mediated by the dendrimers in which electrostatic and lipophilic properties are complementary to the enzyme binding active sites. In contrast, higher generation dendrimers are too large to fit the same binding site within the pocket, suggesting that they interact mainly with the exposed functional groups at the enzyme surface, or exert their effects mainly by modulating other molecular targets. 

## 4. Conclusions

High fidelity coupling of alkynes with azides catalyzed by copper has offered a useful platform in the tailoring and design of multifunctional nanocarriers, and in providing a detailed understanding of timely therapeutic interventions. Articulation of this reaction in a variety of different environments has been the key to implementing the build-up of synthetic architectures, in which participating components with different biological functions are placed at desired locations. Click chemistry has been utilized in developing a variety of multifunctional nanocarriers based on dendrimers and miktoarm polymers. These macromolecules, with their advantageous combination of properties, can be directed towards specific cell organelles, including mitochondria and lipid droplets. Due to ease with which sequential “click” reactions can be performed in these macromolecules, this methodology can be extended to the design of novel nanocarriers with any desired combination of ingredients. It is expected that branched (miktoarm stars) and hyperbranched (dendrimers) architectures will continue to play a pivotal role in biological and medical research and click chemistry will be an essential component in implementing the design of multivalent and multifunctional nanocarriers.

## Supplementary Materials

Supplementary File 1
