# Supplementary material for: Alkyne-Azide “Click” Chemistry in Designing Nanocarriers for Applications in Biology
Source: Molecules. 2013 Aug 8;18(8):9531–49. doi: 10.3390/molecules18089531 (PMC6270461; doi:10.3390/molecules18089531)
Supplement: Supplementary File 1 [file molecules-18-09531-s001.pdf]

Table of Contents Entry

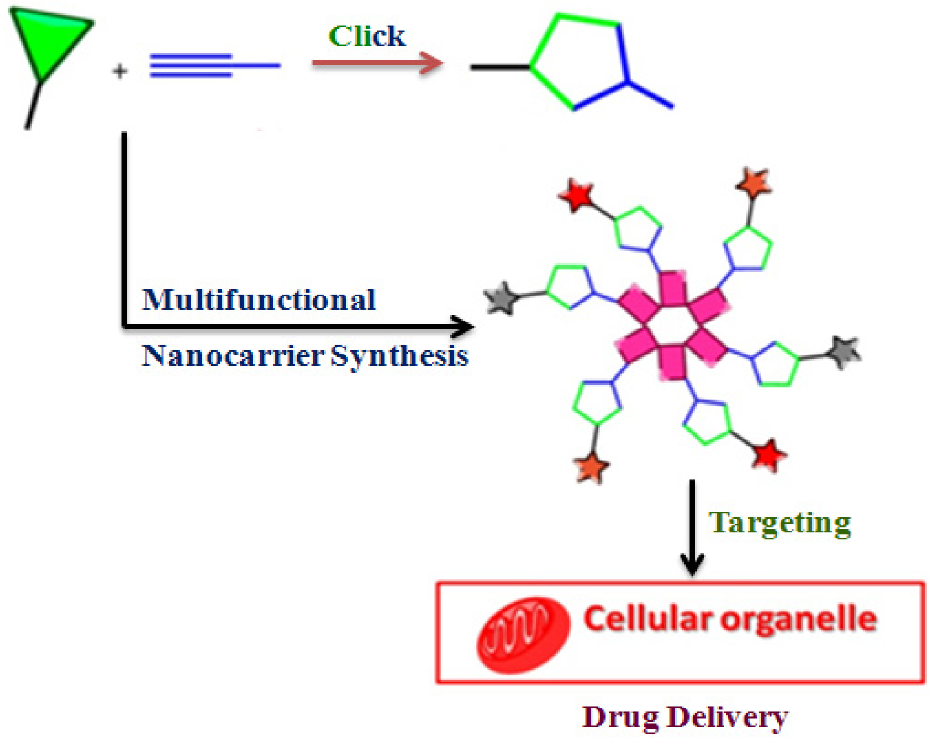

"Click chemistry" provides a versatile platform for tailoring and designing multifunctional nanocarriers with desired physico-chemical properties for targeted delivery of therapeutic agents to specific cell organelles.
